# Highlights from the Eighth International Society for Computational Biology (ISCB) Student Council Symposium 2012

**DOI:** 10.1186/1471-2105-13-S18-A1

**Published:** 2012-12-14

**Authors:** Alexander Goncearenco, Priscila Grynberg, Olga B Botvinnik, Geoff Macintyre, Thomas Abeel

**Affiliations:** 1 Computational Biology Unit, Department of Informatics, University of Bergen, Bergen, Norway; 2 Departamento de Bioquímica e Imunologia, Instituto de Ciências Biológicas, Universidade Federal de Minas Gerais, Belo Horizonte, MG, Brazil; 3 Department of Biomolecular Engineering and Bioinformatics, University of California - Santa Cruz, USA; 4 NICTA, Victoria Research Lab, The University of Melbourne, Victoria 3010, Australia; 5 VIB Department of Plant Systems Biology, Ghent University, Gent, Belgium; 6 Broad Institute of MIT and Harvard, Cambridge, MA, USA

## Abstract

The report summarizes the scientific content of the annual symposium organized by the Student Council of the International Society for Computational Biology (ISCB) held in conjunction with the Intelligent Systems for Molecular Biology (ISMB) conference in Long Beach, California on July 13, 2012.

## About the Student Council and the symposium

The Student Council (SC) is part of ISCB and is led by students and postdocs. Our mission is to nurture and assist the next generation of computational biologists. We offer networking opportunities and soft skill training to scientists in bioinformatics who are in the early stages of their career.

The SC Symposium series has been running for eight consecutive years: Vienna 2011 [1], Boston 2010 [2], Stockholm 2009 [3], Toronto 2008 [4], Vienna 2007 [5], Fortaleza 2006 and Madrid 2005. Every second year when ISMB and ECCB are not jointly organized, an additional symposium is held called the European Student Council Symposium (ESCS). This has been running since 2010 and this year ESCS was held in conjunction with ECCB in Basel.

## Scope and format of the meeting

The Student Council Symposium is a day-long meeting held in conjunction with the ISMB/ECCB conferences every year. The goal of our activities at ISMB is to help fellow students build their career in computational biology. We do this by creating opportunities to meet peers from all over the world, promote the exchange of ideas and provide networking opportunities. The 8th ISCB Student Council Symposium (SCS8) started with a scientific speed dating session in which participants introduced themselves and their science to a new person every two minutes. The traditional scientific component of the meeting consisted of three sessions, each with a keynote talk and several student presentations. In the evening, everybody got the opportunity to present their work during the poster session.

This year’s keynote lectures were kindly delivered by Dr. Robin Dowell (University of Colorado at Boulder), Dr. Matthew Hibbs (University of Maine at The Jackson Laboratory), and Dr. Jonathan Eisen (University of California, Davis). Furthermore, three institutional partners gave short presentations about career opportunities at their respective institute: NICTA (http://www.nicta.com.au), EBI (http://www.ebi.ac.uk) and EMBO (http://www.embo.org).

This year, the symposium received 103 submissions from students. These submissions were peer-reviewed by 56 independent reviewers. Ten abstracts were selected for oral presentation and approximately 50 additional abstracts were accepted for poster presentations. Abstracts of the oral presentations are included in this meeting report. Abstracts of the poster presentations are available online in the symposium booklet (http://symposium.iscbsc.org/content/booklet).

## Keynotes

The day began with a keynote by Dr. Matthew Hibbs, who linked discrepancies between RNA and DNA, known as RNA-editing, to micro-RNA (miRNA) via stem cell development. According to Dr. Hibbs, RNA-editing events lead to novel miRNAs and functional relationship networks in the developing mouse embryo are quite different from cell to cell. He also introduced the StemSight website as a means of visualizing these differences.

After the lunch break, Dr. Jonathan Eisen regaled us with stories of his graduate career, encouraged us to keep our science open-access, take advantage of random opportunities, “what you don’t know can hurt you,” and how phylogeny is a driving force behind bioinformatics. Splits in phylogenetic trees can be found by variation at a single allelic site, and the effects of this SNP may be unknown.

Dr. Robin Dowell closed the session by challenging common ideas about transcription, showing that the mere act of transcription of one gene can block transcription of a gene on the opposite strand. After presenting these results to biologists, Dr. Dowell reported that many approached her and said this transcription-blocking-transcription effect explains this baffling immunoblot, pointing to their lab notebook.

## Student presentations

The first student presentation by Gong et al. [6], followed on nicely from Dr. Hibbs talk by presenting miRNASNP, a database which predicts the effect (loss or gain of function) of single nucleotide polymorphisms (SNPs) within pre-miRNA, mature miRNA, miRNA target sequences and flanking regions.

To find SNPs in miRNA-related regions or in an entire genome, next-generation sequencing is the tool of choice. However, sequencing is a chemical, not digital, process and thus can be noisy. Viswanath et al. [7] tried to address this noise by improving color (and thus base) calling of the SOLiD 5500 sequencer via multiclass support vector machines.

Epigenetics play a critical role in development and disease, and Dinh et al. [8] presented Epi-Letter, an epigenetics visualization tool, presented with published Arabidopsis thaliana chromatin data. Even so, understanding epigenetic effects will remain a challenge.

One of the most commonly described bacterial transcription initiation marker, the Shine-Dalgarno sequence, is complementary to the anti-Shine-Dalgarno sequence within the 16S ribosome. Lim et al. [9] showed degeneration in the anti-Shine-Dalgarno sequence of fifteen out of 1182 bacterial genomes, implying additional, unstudied markers of transcription initiation.

ChIP-seq provides new opportunities to study allele-specific protein-DNA binding, a hard task because only sequence reads mapped to heterozygote SNPs are informative for allelic inference. To better understand allelic variation, Wei et al. [10] presented IASeq, a method for determining allele-specific binding in ChIP-Seq studies by integrating multiple data sets.

Iacucci et al. [11] created ReLiance to predict receptor ligand binding modeled after the same principles as weighted matching algorithms used on online dating sites. Each side of the interaction has a set of criteria, much like the known qualities of a receptor or ligand, but these criteria could be adaptive to the situation.

Recent advances in mass spectrometry technology have improved their usefulness for imaging biological samples. Bemis et al. [12] presented statistic-based signal processing and feature selection methods for desorption electrospray ionization (DESI) imaging mass spectrometry experiments. Their method performs a better spatial segmentation and improves the mapping between morphological and functional structures. The circadian rhythm allows the organism to anticipate and prepare for the changes in the physical environment, in particular the day-night cycle. Nandu et al. [13] reconstructed regulatory networks involved in the cyanobacteria circadian rhythm via a combination of text-mining and expression data.

Gene expression data can be difficult to analyze, especially to find clusters of co-expressed genes. Biclustering of gene expression data is used to discover groups of genes that are co-expressed over a subset of tested conditions. Voggenreiter et al. [14] discuss an exact solution to the bi-clustering problem, BiMax. Their tool is significantly faster than competing algorithms and allows for analysis of hundreds of experiments at a time.

In addition to differential expression analysis, mRNA transcript reconstruction can also be performed from gene expression data. Mangul et al. [15] presented a transcript reconstruction tool based on integer programming, which improves accuracy on previous methods that did not take into account the fragment length distribution.

## Award winners

This year, four awards where giving to the best student presenters of the day, two for oral presentations and two for poster presentations. The abstracts of the poster winners are included in this meeting report. The awards were decided by delegate voting and a judging committee.

The best presentation award winners were Kyle Bemis and runner-up was Oliver Voggenreiter. For the poster awards, first place went to Heewook Lee who presented on detecting structural variants that involve repetitive elements in E. coli [16]. The second prize was given to a jointly presented poster by Ignacio S. Caballero and Gracia Bonilla, who presented the tracking of antiviral responses after infection with Lassa virus [17].

## Student Council activities at ISMB

### Career Central

For the second year running, the Student Council organized a Career Session during ISMB designed to provide students with information to help them make informed decisions about their future careers. We were extremely fortunate to have Dr. Lucia Peixoto, Post-doctoral Fellow at University of Pennsylvania, Dr. Jeroen de Ridder, Assistant Professor at Delft University of Technology, Dr. Yana Bromberg, Assistant Professor, Dept. of Biochemistry and Microbiology at Rutgers University, Dr. Pankaj Agarwal, Director, Systematic Drug Repositioning, Computational Biology at GlaxoSmithKline, and Dr. Michal Linial, Professor at The Hebrew University as part of the discussion panel. The panel members fielded an hour of questions from the audience ranging from how to stay motivated during a PhD through to outlining career options post PhD.

To help job seekers and job advertisers, the Student Council provided an enhanced job advertising process throughout the meeting in the exhibitor hall. Job advertisers could meet with job seekers through our interactive job posting board. Many impromptu interviews were carried out at the Student Council booth during the conference with positive feedback from participants, both interviewers and interviewees.

### Social activities

An important aspect of conferences is social networking. The Student Council has a comprehensive program of social activities outside the conference hours to continue discussions, cement collaborations and exchange knowledge. The daily Social HQ saw up to 50 students meet up over dinner and drinks each night, providing the opportunity for students to network and discuss their scientific and career challenges. On Sunday, the Student Council hosted a social event with free appetizers and fostered social ties with a trivia quiz titled “CLUSTER Your Talent” which challenged delegates’ knowledge of popular culture as well as their expertise in the area of computational biology.

## Conclusions

This year saw a slight decline in the number of submission and participants. We surveyed students who were unable to attend and the most commonly cited reasons were lack of funds and visa issues, reinforcing the need for Travel Fellowships and coordination with ISCB and the host country. Nevertheless, we were once again able to organize a program with three high-quality student presentation sessions, a broad poster session and three invited keynotes. Coupled with the other Student Council activities organized during ISMB, the SC again catered to a large fraction of students in bioinformatics and computational biology.

Looking forward, with SCS8 another success planning will start on SCS9 at ISMB/ECCB 2013 in Berlin, Germany. Further information regarding the Student Council, its events, internships and community, please visit http://www.iscbsc.org.

## References

[B1] Grynberg P Abeel T Lopes P Macintyre G Rubino LP Highlights from the Student Council Symposium 2011 at the International Conference on Intelligent Systems for Molecular Biology and European Conference on Computational Biology BMC Bioinformatics 2011 12 11 10.1186/1471-2105-10-S13-I1 PMC2764124 19840405

[B2] Klijn C Michaut M Abeel T Highlights from the 6th International Society for Computational Biology Student Council Symposium at the 18th Annual International Conference on Intelligent Systems for Molecular Biology BMC Bioinformatcs 2010 11 Suppl 10 4 10.1186/1471-2105-11-S10-O4 PMC2764124 19840405

[B3] Abeel T de Ridder J Peixoto L Highlights from the 5(th) International Society for Computational Biology Student Council Symposium at the 17(th) Annual International Conference on Intelligent Systems for Molecular Biology and the 8(th) European Conference on Computational Biology Introduction BMC Bioinformatics 2009 10 13 10.1186/1471-2105-10-S13-I1 PMC2764124 19840405

[B4] Peixoto L Gehlenborg N Janga SC Highlights from the Fourth International Society for Computational Biology Student Council Symposium at the Sixteenth Annual International Conference on Intelligent Systems for Molecular Biology BMC Bioinformatics 2008 9 10 10.1186/1471-2105-9-S10-I1 PMC3226086 19007430

[B5] Gehlenborg N Corpas M Janga SC Highlights from the Third International Society for Computational Biology Student Council Symposium at the Fifteenth Annual International Conference on Intelligent Systems for Molecular Biology BMC Bioinformatics 2007 8 8

[B6] Gong J Tong Y Zhang H-M Guo A-Y miRNASNP: a database of miRNA related SNPs and their effects on miRNA function BMC Bioinformatics 2012 13 S18 A2

[B7] Viswanath S Yang C Color call improvement in next generation sequencing using multi-class support vector machines BMC Bioinformatics 2012 13 S18 A3

[B8] Dinh HQ Epi-letters - how to describe epigenetic signatures BMC Bioinformatics 2012 13 S18 A4

[B9] Lim K Furuta Y Kobayashi I Unexpected variations in translation initiation machinery BMC Bioinformatics 2012 13 S18 A5

[B10] Wei Y Li X Wang Q Ji H iASeq: integrating multiple chip-seq datasets for detecting allele-specific binding BMC Bioinformatics 2012 13 S18 A6

[B11] Iacucci E Tranchevent L-C Popovic D Pavlopoulos GA Moor BD Schneider R Moreau Y A bioinformatics e-dating story: computational prediction and prioritization of receptor-ligand pairs BMC Bioinformatics 2012 13 S18 A7

[B12] Bemis KD Eberlin L Ferreira C Cooks RG Vitek O Spatial segmentation and feature selection for desi imaging mass spectrometry data with spatially-aware sparse clustering BMC Bioinformatics 2012 13 S18 A8

[B13] Nandu T Pradhan M Palakal MJ Elucidating gene signatures that control the circadian rhythm in cyanobacteria using bioinformatics methods BMC Bioinformatics 2012 13 S18 A9

[B14] Voggenreiter O Bleuler S Gruissem W Exact biclustering algorithm for the analysis of large gene expression data sets BMC Bioinformatics 2012 13 S18 A10

[B15] Mangul S Caciula A Sakarya O Mandoiu II Zelikovsky A An integer programming approach to novel transcript reconstruction from paired-end rna-seq reads BMC Bioinformatics 2012 13 S18 A11

[B16] Lee H Popodi E Foster PL Tang H Detecting structural variants involving repetitive elements: capturing transposition events of IS elements in the genome of Escherichia coli BMC Bioinformatics 2012 13 S18 A12

[B17] Caballero IS Bonilla G Yen JY Connor JH Diagnosing Lassa Virus Infection by Tracking the Antiviral Response BMC Bioinformatics 2012 13 S18 A13

